# The Cumberland Infirmary, Carlisle

**Published:** 1914-10-10

**Authors:** Henry Burdett


### REPORTS ON Hospitals of the United Kingdom.

### THE CUMBERLAND INFIRMARY, CARLISLE—AND CRITICISM.

By SIR HENRY BURDETT, K.C.B., K.C.V.O.

My Report on this institution, published in The Hospital of September 19, 1914, page 677, was written as the result of requests made to me in August and October 1909 by the Chairman of Committee and by an eminent member of the honorary medical staff. The latter enclosed in his letter a block plan of the whole of the infirmary, including the new extensions then in progress. It was not possible to comply with the request made earlier than June 20 last. I have always been interested in the welfare of the Cumberland Infirmary, having known several of its officers who have made high reputations and have risen to important positions in the hospital world. This feeling, and the letters just referred to, made me, if possible, more than usually anxious to make my Report contribute to the bringing up of this important institution to the standard of an up-to-date modern hospital. I pointed out that, "structurally the institution has been transformed for the better," but "administratively it has been changed sadly for the worse." That is what I found in the fewest possible words. I have now almost completed the inspections of the voluntary hospitals throughout the country, and my Reports, of which that on the Cumberland Infirmary is one, have been welcomed by the managers and have resulted almost everywhere in action which has caused the chief voluntary hospitals to approach, if they have not everywhere reached, the standard of a modern up-to-date hospital. It remains to be seen whether the committee and governors of the Cumberland Infirmary will unite in an effort to make the administration of the infirmary up to date and smartly efficient.

In my Report I suggested there was urgent need for an independent investigation at Carlisle, and the letter from Dr. Lediard, one of the honorary medical staff, which appears on the next page, confirms the practical value of this recommendation. There is evidence that Dr. Lediard and the infirmary authorities do not appreciate the responsibility of providing adequate recreation-room accommodation for the large staff of laundrymaids they employ. Further, that they do not realise the effects of the working of the present system of administration, which I emphasised and have good grounds to maintain. The question of a garden and all I urged in regard to it are not matters of appreciable cost, but of sound management and of lost opportunities for expediting the recovery of the patients. The Cumberland Infirmary contains 140 beds, and Dr. Lediard's description of it as "a small provincial hospital" is, therefore, inaccurate. My criticisms rest upon the standard attained by a modern up-to-date hospital, whilst Dr. Lediard appears to be content with something infinitely inferior to it.

Dr. Lediard's letter is no answer to my Report. He accuses me of "certain inaccuracies which mar the trustworthiness of his [my] statements," to wit, that the reconstruction has included (1) a new ophthalmoscopic room, and (2) a new clinical room for ward work. He describes the former as a completely equipped ophthalmic department, and declares the latter has not yet been acquired. I must remind Dr. Lediard that the lithograph plan supplied to me from the infirmary contains the additions and alterations signed by the architect, and states on its face that these include: D, a new clinical room; and K, a new ophthalmoscopic room. The inaccuracies, if inaccuracies there be, are, therefore, not mine, and if they mar the trustworthiness of any statements, as Dr. Lediard declares, they cannot be the statements in my Report.

For the rest Dr. Lediard ignores altogether the pertinent questions which my Report contained, and contents himself by writing at length and at large in the apparent attempt to draw a herring across the scent. He declares and continuously insists that "we know how to adapt our expenditure to our means," an assertion which, for reasons set forth clearly in my Report, I beg leave to question. He has evidently to learn, with others, the importance in hospital administration to remember "there is that spendeth and yet increaseth."

In endeavouring to secure a thorough and impartial inquiry in the best interests of the future of Cumberland Infirmary, I am handicapped by the apparent general apathy, due possibly to the absence of a local daily newspaper. Dr. Lediard inferentially implies that I have attacked the committee of management, and have found something wrong with the matron, Miss Sylvia Parker. So far from attacking the committee, I have merely urged them to make certain changes in the internal management, and to take steps to secure an independent report by a hospital expert of wide experience. Miss Sylvia Parker has had a most difficult task, and has shown much devotion in her efforts to secure good management under conditions which I venture to think ought never to have existed. They, too, should have been removed before she entered upon her office.

For the rest I commend the present administrative working of the Cumberland Infirmary to the attention of the governors and the committee of management, with the expression of an earnest hope that they will adopt the course recommended in my Report, because it should result in increasing its resources and bringing its administration in future nearer to the standard of a modern up-to-date hospital.

